# Perspectives on Self-Management and Meditation: A Qualitative Study of Adolescents With Type 1 Diabetes Mellitus and Their Parents

**DOI:** 10.7759/cureus.70019

**Published:** 2024-09-23

**Authors:** Ejura Y Salihu, Asma M Ali, Judith H Hassan, Deborah T Joseph, Betty Chewning

**Affiliations:** 1 Department of Family Medicine and Community Health, University of Wisconsin-Madison, Madison, USA; 2 Department of Health Sciences and Social Work, Western Illinois University, Macomb, USA; 3 Department of Social and Administrative Sciences, University of Wisconsin-Madison, Madison, USA

**Keywords:** diabetes distress, diabetes self-management, perceived barriers, adolescents, meditation, type 1 diabetes mellitus (t1dm)

## Abstract

Background: One in three adolescents with type 1 diabetes mellitus (T1DM) experiences diabetes distress, which predicts poor self-management and glycemic control. Mindfulness-based interventions such as meditation have been associated with reduced psychological distress and health outcomes in different populations. This study explores the psychosocial barriers and facilitators of diabetes self-management and beliefs about meditation practices.

Methods: Eight adolescents aged 15-19 who had been diagnosed with T1DM for more than a year were invited to participate in a 40-60-minute semi-structured one-on-one interview. Their parents were also invited to participate in the study. Three of the eight parents invited were able to participate in the study. Participants were asked about perceived psychosocial barriers and facilitators of diabetes self-management and their beliefs about meditation as a tool for addressing some of the psychosocial barriers to self-management. Data were analyzed using NVivo 10 (QSR International, Melbourne, Australia). Conventional content analysis was conducted based on an inductive coding approach.

Results: Adolescents with T1DM had similar psychosocial challenges with managing T1DM, including high levels of diabetes distress and forgetfulness due to competing demands on their time. They also noted similar facilitators to effective self-management, such as the presence of family and peer support. Acceptance of T1DM diagnosis and personal commitment to self-management were also indicated as common facilitators of self-management. Adolescents with T1DM and parents of adolescents with T1DM believe that meditation can play a positive role in T1DM self-management by reducing diabetes distress and improving mental health and overall well-being.

Conclusion: Results suggest that adolescents with T1DM and parents of adolescents with T1DM believe peer and family support is crucial to diabetes self-management. They also noted that diabetes distress and forgetfulness are primary barriers to self-management. Participants also see a potential for meditation to help manage general stress and diabetes distress, thereby aiding self-management. Further research is needed to explore meditation-based interventions to reduce diabetes distress in adolescents diagnosed with T1DM. The findings from this study can inform the development and implementation of meditation-based interventions that integrate family and peer support to reduce diabetes distress and enhance self-management in adolescents with T1DM.

## Introduction

Adolescence can be a stressful developmental period characterized by abrupt hormonal and physical changes that can impact social and emotional well-being [1-3]. During this phase, adolescents also navigate changing relationships with family members and peers while developing their identity and autonomy [4,5]. Adolescents with chronic health conditions like type 1 diabetes mellitus (T1DM) face additional burdening stressors related to disease self-management [6-8]. T1DM is a chronic auto-immune disease that requires a complex self-management regimen, including monitoring blood glucose, insulin administration, calorie counting, and regular exercise [6-9]. The burden of self-management, dealing with diabetes-related complications (or the possibility of their occurrence), and handling challenging social circumstances can cause significant emotional distress [10-12].

Diabetes distress is often described as feelings of frustration or exhaustion with self-management tasks and significant anxiety about developing diabetes complications [12-14]. Several studies show that adolescents with high levels of diabetes distress are less likely to engage in self-management tasks, such as blood glucose monitoring, and are more likely to develop diabetes complications [9,14]. Despite the documented high prevalence of diabetes distress in adolescents and its impact on health behaviors and outcomes, standard diabetes care in clinics does not adequately address it in adolescents with T1DM [13,15,16].

Meditation is a subset of ancient self-regulation techniques used to achieve relaxation, mental focus, and moment-to-moment awareness [17,18]. Though the origin of meditation can be traced to ancient Buddhism, there has been a surge in meditation practice in popular culture and clinical psychology research [18-24]. One of the key elements in most meditation practices is the deliberate direction of attention toward sensory experiences (e.g., breathing) or mental processes such as thoughts [25]. Regardless of the kind of meditation, studies show that during meditation, awareness is heightened, which can lead to a better sense of self and well-being [17,26]. The practice has also been linked to reductions in stress, anxiety, and depression levels in different populations [17]. Thus, meditation-based interventions can be potentially effective in improving an individual's experience [23,27] and perception of the burden of diabetes [23,28].

Few studies examine how psychosocial factors affect adolescents with diabetes [8-11,15,29]. Even fewer studies explore how meditation can help adolescents with diabetes distress [15,22,23,27,28,30]. These limitations of previous studies highlight the need for a more thorough qualitative investigation into the psychosocial barriers and facilitators of self-management among adolescents with T1DM. It also highlights the need to explore the potential role that meditation can play in addressing some of the psychosocial barriers to T1DM management.

Objective

To explore the perspectives of adolescents with T1DM and their parents on the psychosocial barriers and facilitators of diabetes self-management and their beliefs about meditation as a tool for addressing some of the psychosocial barriers to self-management.

## Materials and methods

Using a purposive sampling technique, eight adolescents living with T1DM were recruited from a pediatric clinic in a Midwestern state in the US. Adolescents were invited to participate in the study if they met the following criteria: (1) 15-19 years old and (2) diagnosed with T1DM for more than one year. Parents of these adolescents with T1DM were also invited to participate in the study. Of the eight parents invited, three agreed to participate in the study. To ensure that diverse perspectives were included in the study, experience with meditation (or lack thereof) was not a criterion considered during participants’ recruitment.

Data collection

An experienced qualitative researcher (E.Y.S.) conducted 40-60-minute semi-structured one-on-one interviews with each participant between September 2021 and October 2022. As a researcher without T1DM, she recognized that her outsider status could influence how she interpreted the experiences of adolescents with the condition. To mitigate this, she created a safe space for participants to share their experiences and sought input from healthcare professionals who work with this population. The interviews were conducted via a secure web-based meeting platform, WebEx (Cisco Systems, Inc., San Jose, USA). Separate interview guides were used for each group of participants (adolescents and parents). Interview guides are provided in the Appendices. Individual interviews are ideal for eliciting in-depth views about sensitive topics that participants may otherwise be uncomfortable sharing in a large group [31]. Eleven interviews were audio-recorded and transcribed verbatim, with identifying information redacted.

Data analysis

Four researchers with training in qualitative research conducted conventional content analysis using an inductive coding approach [31-35]. The data was managed and analyzed on NVivo 10 (QSR International, Melbourne, Australia). The researchers read the interview transcripts line-by-line, independently coded two transcripts as a test set, and met to compare their codes before agreeing on a codebook. Then, they independently coded all the transcripts using the codebook. Afterward, the researchers consolidated their codes into categories. The categories were then organized into relevant themes [34]. Lincoln and Guba’s criteria for establishing trustworthiness in qualitative research were used to ensure rigor [35]. Multiple coders analyzed the data to get rich and robust interpretations. Thick descriptions of the method, data collection, and analysis have been provided to aid the transferability of findings to similar contexts. The researchers conducted member checking by sharing the results with all the participants to ensure the accuracy of quotes and interpretation [35].

## Results

The adolescent participants in this study consisted of eight individuals ranging in age from 16 to 18. Five of the eight adolescents interviewed were 17 years old. All of the adolescents interviewed identified as White, and 50% of the participants identified as female.

For the parent group, three parents participated in the study. All of the parents interviewed were White females. Each parent was linked to their respective adolescent participant, providing additional contextual data for the adolescent results. Participants' characteristics are summarized in Tables 1-2.

**Table 1:** Demographic Information of Adolescent Participants

Participant ID	Age	Race	Sex
Adolescent 1	17	White	Male
Adolescent 2	18	White	Female
Adolescent 3	17	White	Female
Adolescent 4	16	White	Male
Adolescent 5	17	White	Female
Adolescent 6	17	White	Male
Adolescent 7	18	White	Female
Adolescent 8	16	White	Male

**Table 2:** Demographic Information of Parent Participants

Participant ID	Race	Sex
Parent 1 (Parent of Adolescent 1)	White	Female
Parent 2 (Parent of Adolescent 2)	White	Female
Parent 3 (Parent of Adolescent 8)	White	Female

Participants' experiences regarding diabetes self-management and their perceptions about meditation were conceptualized into three domains: psychosocial barriers to self-management, psychosocial facilitators of self-management, and perceived benefits of meditation. A summary of domains and themes is presented in Table 3.

**Table 3:** Domains and Themes Related to Psychosocial Factors and Perceived Benefits of Meditation in Diabetes Self-Management

Domains	Themes
Psychosocial barriers to self-management	Diabetes distress
	Forgetting to perform diabetes self-management tasks due to competing demands on time
Psychosocial facilitators of self-management	Family support
	Peer support from diabetes camps
	Acceptance of diagnosis and need for self-management
Perceived benefits of meditation	Meditation can help relieve acute and chronic diabetes distress
	Meditation can help improve acceptance of diagnosis and self-management
	Meditation can help improve focus and overall emotional well-being

Psychosocial barriers to self-management

Adolescents with T1DM experience psychosocial challenges that impede their ability to perform self-management tasks. These challenges include diabetes distress and forgetting to perform self-management tasks due to competing demands on time.

Theme 1: Diabetes Distress

Frustration, exhaustion, and being overwhelmed with diabetes self-management tasks - feelings associated with diabetes distress - were noted as leading barriers to self-management in adolescents with T1DM by both adolescents and parents.

"Well, I have had diabetes for 11 or 12 years, so I have a pretty good grip on it, but last year, I did a crappy job taking care of myself because I was just kinda sick of it and done with it." - Adolescent 5

"When you have diabetes, you have to make more decisions a day than a typical person. You have to be constantly thinking about food that you are being given, and then making decisions about how much insulin to give. The decision fatigue is real. There is exhaustion that comes from managing the disease all the time." - Parent 1

Theme 2: Forgetting to Perform Diabetes Self-Management Tasks Due to Competing Demands on Time

Another barrier to optimal self-management was forgetting to do self-management tasks due to competing demands on time.

"If I am at work and it is really busy, I don't have time to check my blood glucose for a minute. It gets really hard trying to do that because my work's counting on me to be doing what I'm there to do." - Adolescent 1

"Sometimes [at work], when I am stressed, I forget to do stuff, so I forget the [insulin] dose." - Adolescent 8

Parents also echoed the adolescents' concerns about competing demands on time and forgetfulness being key barriers to self-managing diabetes. One parent said: "Trying to get her to remember now because she does not always have extra supplies on hand. She started a new job this summer, and twice, she had to call us and ask us to bring her supplies because her insulin ran out or she had forgotten her extra set … that has been the hardest part: trying to get her to make sure she takes extra supplies with her when she goes places." - Parent 2

Psychosocial facilitators of self-management

Three themes emerged as facilitators of self-management: family support, support from peers diagnosed with T1DM, and personal behavioral facilitators (e.g., acceptance of T1DM diagnosis and need for self-management).

Theme 1: Family Support

Family support was a significant facilitator of self-management. All the adolescents and parents believed that parents played a crucial role in adolescents' self-management of T1DM.

"For a while, it was my mom. She would be on top of me, saying, 'Hey, you are about to get low. I need you to do your blood glucose check,' but after a while, with me getting older, I would blow her off, but I would still do it." - Adolescent 1

"I usually try to make sure that she gets up and eats breakfast to help get her blood glucose level more stable. I still check the [glucose monitoring] app, especially if she is doing more active things on the weekend, like socially. I will check to make sure that her blood glucose is okay. If I see that something is either getting too high or too low, I text her or check in with her and ask her if she has taken a look, so I am observing and prompting her to do things I think she should." - Parent 2

Theme 2: Peer Support From Diabetes Camps

Both parents and adolescents reported the perceived benefits of having peer support as complementary to the support provided by family. While parents provide hands-on support, especially around the initial diagnosis, there was agreement that peer support from others with T1DM also plays a valuable role in long-term diabetes management.

"Every year, I go to this diabetes camp where there is a whole ton of [kids with diabetes] shoved in a little campground. That is always super helpful because you get to talk to other kids [with diabetes], which makes you feel like you are not the only one going through it … feels like a community." - Adolescent 5

"My son went to a diabetes camp for a couple of years…and that is where he finally started to do the set changes and things themself. He was much more willing to hear it from someone other than me…someone who understands what he is going through." - Parent 1

Theme 3: Acceptance of Diagnosis and Need for Self-Management

The key facilitators of self-management were acceptance of diagnosis and commitment to managing the disease.

"I feel like you need to be able to cope and accept it and not try to avoid it. Otherwise, you are not going to take good care of yourself. It is a part of your life but not your life. So you need to acknowledge it but not let it control you." - Adolescent 3

"I think maturing and realizing how important it is to take care of it [T1DM] well-thinking about the future when I will be living on my own. I will have to be able to do all this alone without any help from my parents … at least, pretty much no help from my parents. That has made me want to make sure that I am able to manage it well." - Adolescent 4

Perceived benefits of meditation

Participants indicated many benefits of meditation as a valuable practice for managing stress and improving memory, focus, and self-acceptance, which would help in T1DM self-management.

Theme 1: Meditation Can Help Relieve Acute and Chronic Diabetes Distress

Participants believe meditation can aid stress management. They specifically stated its potential to help manage both acute and chronic diabetes distress, which will, in turn, aid self-management.

"Meditation would help after a hypoglycemic or hyperglycemic event, help me calm down, and remind me to stop stressing out about it so much." - Adolescent 7

"I think meditation will help with stress. There is a lot of anxiety with feeling like it is [management of T1DM] too much. I think it could maybe help them [adolescents with T1DM] get in a better frame of mind, maybe just be able to release stress for a while and feel a little bit better equipped to manage it." - Parent 3

Theme 2: Meditation Can Help Improve Acceptance of Diagnosis and Self-Management

Participants also reported that meditation could help adolescents become more aware of the present moment and less judgmental of themselves when things go wrong, leading to more self-acceptance.

"With diabetes care, there are a lot of elements of judgment. You chose to eat the entire bag of candy, and now you are in this mess. There is just tons of judgment in there. I think that if you were actively using meditation, it would give you the tools to say, 'Well, that was the decision I made, and now here is the situation I am in, so what do I need to do to clear my mind to focus and solve the problem?' It [meditation] helps you clear your mind so you can focus on that [self-management] task and accept it." - Parent 1

Theme 3: Meditation Can Help Improve Focus and Overall Emotional Well-Being

All the participants stated their belief in meditation as a helpful practice for adolescents who wish to improve their mental focus and overall well-being. Many adolescents wanted to meditate more often to "be less stressed and focus better."

"I think meditation is such a reflective practice that can put you in a different mindset where you can see things in a better light." - Parent 2

## Discussion

Effectively managing T1DM requires adhering to multiple self-management practices, including regularly monitoring blood glucose levels, administering insulin accurately, tracking calorie intake, and engaging in regular physical exercise [6,9]. The burden of self-management and the risk of diabetes-related complications are associated with diabetes distress in adolescents [10,11]. This qualitative study explored the perspectives of adolescents with T1DM and their parents on the psychosocial barriers and facilitators of diabetes self-management and their beliefs about meditation as a tool for reducing the psychosocial barriers to self-management.

Three of the eight adolescents had significant experience with meditation (more than three months of experience with meditation), and four had recently tried at least one form of mindfulness exercise (e.g., focusing on breathing or counting slowly). The high number of participants who reported some experience with meditative practices corresponds with previous studies that show an increase in overall meditation practice among US adolescents [36] and adults [37].

Barriers to self-management

Two key barriers to self-management were noted. The first factor is diabetes distress, characterized by a sense of being overwhelmed by the demands of self-management. In our study, adolescents and their parents described feelings of frustration, exhaustion, and being overwhelmed by the multiple self-management routines. Therefore, our finding underscores the significance of these emotional experiences in shaping self-management behaviors [38-40].

Another common challenge noted was the tendency to forget to perform self-management tasks due to various demands on adolescents' time. Both adolescents and parents admitted that social and work obligations often eclipsed the need to self-monitor blood glucose levels and administer insulin. The stress associated with work-related responsibilities also compounded their challenges, causing lapses in self-management. This result aligns with a previous study by Hung et al. [41], which found that adolescents with high workloads struggled with completing diabetes self-management routines. Adolescents also expressed psychological difficulties managing their own care, including a dread of needles, forgetting to take their insulin, feeling ashamed, and thinking that having diabetes was a burden in their lives. This result is supported by previous studies on the challenges adolescents with T1DM face [42,43].

Psychosocial facilitators

Participants in this study also identified several psychosocial facilitators that contribute to effective self-management. All participants stated that parents play a pivotal role in self-management because they constantly encourage, remind, and assist their children with diabetes-related tasks like food preparation and buying and keeping insulin supplies. Adolescents said they felt seen and heard when they had a platform to hear the experiences of other adolescents living with T1DM and share coping strategies with each other. Furthermore, the opportunity to share and learn from others improved their self-esteem and self-efficacy. Parents also stated that their children were more likely to accept self-management advice from their peers. This result is consistent with previous literature that shows that family and peer support is crucial to ensuring adolescents with T1DM feel supported in managing T1DM [44,45].

One adolescent in our study reported their acceptance of T1DM diagnosis and knowledge of diabetes complications such as amputation and death were vital in their determination to continue self-management tasks even when exhausted. This finding suggests that while anxiety surrounding diabetes complications is prevalent in adolescents with T1DM and is a significant barrier to self-management [11,12], knowledge of diabetes and diabetes-related complications may also improve self-acceptance and willingness to perform diabetes self-management tasks.

Perceived benefits of meditation

Adolescents and their parents believe meditation can help relieve acute diabetes distress by calming the mind after blood glucose events. One adolescent believed that regular meditation could prevent chronic diabetes distress by helping them accept the T1DM diagnosis and be able to continue self-management tasks even in stressful situations. All the participants believed meditation practices could help adolescents be more present and aware and cultivate a non-judgmental attitude towards themselves, aiding in problem-solving when faced with diabetes-related stressors and enhancing their emotional well-being. This result is validated by previous studies [18,19,23,46,47], which showed that mindfulness interventions can potentially improve psychological well-being and other health outcomes. Given the evidence that meditation reduces psychological distress in previous studies and that the participants in this study have positive expectations of meditation, it is worth exploring more fully and systematically the impact of meditation on diabetes distress in adolescents living with T1DM. To gain a deeper understanding of how meditation can impact diabetes distress and self-management, future research should focus on individuals with limited or no prior experience with meditation.

Limitations

Purposive sampling was utilized for interviews, which allowed data to be gathered from individuals most impacted by T1DM - adolescents with T1DM and their parents. However, this study has some limitations. Participants were recruited from one clinic in one Midwestern state in the U.S. Consequently, all participants were already receiving care and identified as non-Hispanic Whites. The sample size for parents was also small, leading to a narrow view of parents' perspectives. Many of the parents contacted to participate in the study were unable to participate because of schedule conflicts. Despite these limitations, this study provides relevant preliminary information on the perspectives of adolescents with T1DM and their parents, which reinforces the high level of stress adolescents with T1DM face and the potential value of meditation-based interventions to reduce diabetes distress in adolescents.

## Conclusions

Study findings highlight the significant psychosocial challenges that adolescents with T1DM face and the urgent need for accessible peer-led and/or family-driven interventions that improve well-being and health outcomes in this population. Participants believe that meditation has immense potential to help manage both general stress and diabetes-related distress, which in turn can improve T1DM self-management. The findings from this study can inform the design and implementation of meditation-based interventions that integrate family and peer support to reduce diabetes distress, improve well-being, and enhance self-management in adolescents with T1DM.

## Appendices

Interview protocol for adolescents with type 1 diabetes mellitus

Thank you again for agreeing to be interviewed today. My name is ______. I am a student at the ________. This project explores barriers and facilitators of type 1 diabetes self-management among adolescents and beliefs about the potential role of meditation in diabetes self-management. To help us, we are starting by getting a picture of everyone's typical weekday and weekend and seeing how meditation might fit into your schedule. We are also interested in whether you think meditation might be helpful for some of the challenges in caring for diabetes. Before we begin, do you have any questions you would like to ask about the project? I want to confirm your consent to participate in this research. Is that all right?

I would like to start by asking you a few questions about your typical day. You can choose not to answer any of the questions and can stop the interview at any time.

If it is OK with you, I will record our discussion today so I don't miss any of your important comments. Your comments will be strictly confidential, and if we write any reports or publications about this study, we will not use your name. Would that be all right with you?

Let's talk about your typical day during the weekdays.

1.     If you think of a typical day, what do you do each weekday, including taking care of your diabetes?

a.     Is there anything else you'd do on a typical day?

b.     Can you say more about … ?

2.     And now, what do you do on a typical weekend day, including taking care of your diabetes?

a.     Is there anything else you'd do on a typical day?

b.     Can you say more about … ?

Now, I'd like to talk about apps and Meditation

1.     Do you ever use lifestyle apps on your phone?

a.    What types of lifestyle apps have you used?

b.    Do you use any of them at a regular time, or does it vary according to the day and your needs?

c.    When are you most likely to use them?

2.    Have you ever used an app to meditate?  Yes,     No

IF YES: Which app? How were you introduced to it?

3.     If you haven't used an app to meditate, have you ever meditated?

IF YES: Can you tell me about that experience?

IF YES: In general, how did you feel about meditation?

So we've talked about a typical day and meditation. Let's talk about about your diabetes and meditation.

1.      What are some of the most challenging things about managing your diabetes?

2.     To what extent is this stressful to you?

3.     What most helps you now to manage your diabetes?

4.     You mentioned that (x) helps you manage your diabetes. What else, if anything, might make it easier to manage your diabetes?

5.     Do you have any sense of whether or how using a meditation app might either help or hinder taking care of your diabetes?

Probe: Can you talk more about that? Why is that?

6.     Do you have any sense of whether or how using a meditation app might either help or hinder your emotional wellbeing?

Probe: Can you talk more about that? Why is that?

Closing remarks

Thank you again for agreeing to be interviewed today. Before we end this meeting, is there anything else you would like to share about how you manage your diabetes or your views about meditation?

Interview protocol for parents/caregivers of adolescents with type 1 diabetes mellitus

Thank you again for agreeing to be interviewed today. My name is ______. I am a student at the ________. This project explores barriers and facilitators of type 1 diabetes self-management among adolescents and beliefs about the potential role of meditation in diabetes self-management. To help us, we are starting by getting a picture of everyone's typical weekday and weekend and seeing how meditation might fit into your schedule. We are also interested in whether you think meditation might be helpful for some of the challenges in caring for diabetes. Before we begin, do you have any questions you would like to ask about the project? I want to confirm your consent to participate in this research. Is that all right?

If it is OK with you, I will be recording our discussion today, so I don't miss any of your important comments. All your comments will be strictly confidential, and if we write any reports or publications about this study, we will not use your name. Would that be all right with you?

I’d like to learn a bit more about you. 

1.     How old is your child/ward and when were they diagnosed with type 1 diabetes?

2.     If you think of a typical day, what do you do each weekday, including helping your child/ward take care of their diabetes?

3.     Is there anything else you'd do on a typical day?

Follow up: Can you say more about … ?

And now, what do you do on a typical weekend day, including helping your child/ward take care of their diabetes?

5.     Is there anything else you'd do on a typical day?

Follow up: Can you say more about … ?

6.    What are some of the most challenging things about helping your child to care for their diabetes?

7.     On a scale of 1 to 10, how stressful is this to you?

Probe: Can you talk more about that?  Why is that?

8.     What most helps you now to help your child manage the challenges?

You mentioned that (x) helps you manage the challenges, what else, if anything, might make it easier to manage?

9.     What are your thoughts about stress management for young people with diabetes?

10.    What are your thoughts about stress management for parents of young people with diabetes?

Now I'd like to talk about apps and meditation

11.     Have you ever used an app to meditate? 

IF YES: Which app? How were you introduced to it?

If you haven't used an app to meditate, have you ever meditated?

IF YES: Can you tell me about that experience?

12.    In general, how did you feel about meditation?        

13.    What do you think would be the impact of meditation on how adolescents between the ages of 10 and 19 take care of diabetes?

Probe: Can you talk more about that?  Why is that?

a.     What are your thoughts on adolescents with diabetes using meditation apps?

b.     What are the potential barriers to their use of the app?

c.     What can be done to reduce these barriers?

14.    How would you describe emotional wellbeing in adolescents with type 1 diabetes?

15.     Do you have any sense of whether or how using a meditation app might impact their emotional wellbeing?

Probe: Can you talk more about that?  Why is that?

Now let’s talk about you. Do you have any sense of whether or how using a meditation app might impact your ability to help your child/ward care for their diabetes?

16.     Do you have any sense of whether or how meditation might impact your emotional wellbeing?

17.     What are your thoughts on introducing a meditation session at places that adolescents with diabetes frequently go to, e.g., diabetes camps?

Follow up: Can you suggest other potential places we can introduce meditation to adolescents with type 1 diabetes?

18.     Would you be willing to be part of a study examining the impact of meditation on diabetes related stress among children with type 1 diabetes and their parents?

Closing remarks

Thank you again for agreeing to be interviewed today. Before we end this meeting, is there anything else you would like to share about diabetes management or your views about meditation?
